# O7-3 Young people Living, learning and growing - the impact of a year on independent boarding schools

**DOI:** 10.1093/eurpub/ckac094.051

**Published:** 2022-08-29

**Authors:** Lise Elkrog-Hansen, Thomas Skovgaard

**Affiliations:** Research and Innovation Centre for Human Movement and Learning (FIIBL), University of Southern Denmark, Odense, Denmark

**Keywords:** Independent boarding schools, physical acitvity, personal development, community, social interaction

## Abstract

**Background:**

This paper outlines a mixed methods research study conducted at three independent boarding schools (a uniquely Danish type of residential setting for young people between the ages of 14 to 18) that all focus on sport and physical activity. In total independent boarding schools attracts more than 30.000 students per year, which makes it a very popular choice for young people. Interestingly, this particular type of schools have not been researched much.

This study aims to provide an insight into the special qualities of Danish independent boarding schools and explore how a focus on sport and physical activity in relation to personal development of young people plays a role at three of these schools.

**Methods:**

The analyses are based on an online survey distributed to 1020 students at the participating schools in March 2019. In total, 865 students answered the survey. The purpose of the survey was to collect quantitative data on background issues, the students' motives for choosing independent boarding school life and to gain an insight into what the students' perceived as the special qualities of living on such a school - typically for one year. The quantitative findings are enriched by empirical findings and conclusions from qualitative data generated via six MSc theses.

**Results:**

The survey results show that students to a great extent choose an independent boarding school based on the sports program (73%), facilities (69%) and sport performance levels (51%). The students point out getting new friends (92%), being a part of a community (92%), learning to collaborate (91%) and solving conflicts (88%) as some of the special qualities of life at independent boarding schools. The qualitative analyses reveals that sense of community, social interaction with fellow students and the relationship between teachers and students are perceived as the main qualities of this type of school life. Furthermore, the analyses points out that sport and physical activity can both hinder and promote qualities as the ones mentioned.

**Conclusion:**

The study contributes to new and important insights into a popular and unique type of school in Denmark, but more research is undoubtedly needed.

